# Developing a New Neuromodulation Treatment Pathway for the Treatment of Depression

**DOI:** 10.1192/bjo.2022.325

**Published:** 2022-06-20

**Authors:** Andreas Papadopoulos, Nathan Maynard, Samuel Lawton, Tiff Earle, Stephen De Souza, Sarah Oke, Jane Yeandle

**Affiliations:** Somerset NHS Foundation Trust, Somerset, United Kingdom

## Abstract

**Aims:**

To ensure an appropriate treatment pathway is available to patients diagnosed with Depression a STEPPED care model where different types of intervention are offered depending on the patient's experienced severity of Depression. However, a great percentage of patients continue to experience disabling symptoms and fall into the Treatment Resistant Depression (TRD) category. There was a need to review the options available within local Mental Health Services (MHS) for the treatment of patients with depression and TRD.

**Methods:**

A new clinical pathway was designed to provide access to patients to emerging treatments, such as EsKetamine, Vagal Nerve Stimulation (VNS) and repetitive Transcranial Magnetic Stimulation (rTMS). After calculating the local impact of depression to patients, trust resources and society we extrapolated our calculations to neighbouring Trusts covering the South West. A newly developed business plan demonstrating the need for new treatment options and the benefits, financial and otherwise, was presented and underwent various approvals levels by the Trust Governance and Executive Committees and local commissioning groups, before being able to proceed. Within the original business plan, rTMS and VNS were offered in addition to the existing ECT as parts of a new treatment pathway. We are now in the process of incorporating EsKetamine and Transcranial Direct Current Stimulation (tDCS). The clinic was set up in March 2020, just at the beginning of the pandemic, which halted operations for quite a few months before being able to resume recently

**Results:**

We have managed to treat patients with both VNS and rTMS from our Trust, as well from surrounding areas. Patients have already experienced benefits in the recovery from symptoms. The new service has provided another line of support for colleagues in offering bespoke treatment plans to their patients and patients have appreciated having access to new non-traditional treatment options.

**Conclusion:**

Although there has been a primary result in improving patient care, income generation is also possible by positioning the Trust in the forefront of new therapeutics and allowing the provision of service to expand to neighbouring Trusts and private patients. Other benefits are associated with the Trust's reputations and kudos being enhanced and include the forging of new pathways within the developing alliance with General Hospitals, increased ‘visibility’ for training and research opportunities, improved patient satisfaction and improved CQC standing.

